# Ineffectiveness of KeraVio Treatment with Violet Light-Emitting Glasses Without Riboflavin Drops for Progressive Keratoconus

**DOI:** 10.3390/jcm14030773

**Published:** 2025-01-24

**Authors:** Hidenaga Kobashi, Takashi Kumanomido, Takeshi Ide, Naoko Kato, Jun Shimazaki, Motozumi Itoi, Kazuo Tsubota

**Affiliations:** 1 Tsubota Laboratory, Inc., Tokyo 160-0016, Japan; 2 Department of Ophthalmology, School of Medicine, Keio University, Tokyo 108-0073, Japan; 3 Kumanomido Eye Clinic, Tokyo 107-0052, Japan; 4 Tokyo Vision Eye Clinic, Tokyo 100-0005, Japan; 5 Department of Ophthalmology, Tokyo Dental College Ichikawa General Hospital, Chiba 272-0824, Japan; naokato@bc.iij4u.or.jp (N.K.);; 6 Itoi Eye Clinic, Tokyo 150-0043, Japan; itoi@eyeacademy.net

**Keywords:** keratoconus, corneal cross-linking, violet light, riboflavin

## Abstract

**Background**: KeraVio, a portable corneal cross-linking (CXL) treatment modality involving the use of violet light (VL)-emitting glasses, was originally used in conjunction with topical transepithelial riboflavin (BJO 2021). However, after our preliminary findings of endogenous riboflavin in the human cornea and the observation that the relatively low intensity of VL irradiation increased corneal stiffness in porcine corneas (TVST 2021), we evaluated the clinical results of KeraVio without riboflavin drops. **Methods**: Patients with progressive keratoconus were enrolled and randomly divided into a VL irradiation alone group (Group 1) and a no irradiation group (Group 2; control group) (jRCTs032190267). The eyes were exposed to VL (375 nm, irradiance 310 μW/cm^2^)-emitting glasses for 4.5 h daily for 6 months. **Results**: The mean changes in the maximum keratometry value (Kmax) from baseline to 6 months were 0.94 ± 2.65 diopters (D) and 1.76 ± 2.75 D in Group 1 and Group 2, respectively (*p* = 0.705). **Conclusions**: No differences were found between patients who did and did not receive VL irradiation in terms of the clinical outcomes of keratoconus. VL irradiation alone likely did not halt keratoconic progression, and the administration of riboflavin was necessary.

## 1. Introduction

Keratoconus is a progressive disorder that frequently involves asymmetric, inflammatory corneal thinning and is characterized by changes in the structure and organization of corneal collagen [1]. This disease induces the progression of corneal thinning, progressive myopia, and irregular astigmatism. Keratoconus has traditionally been treated with corneal transplants to correct vision [2]. Even today, patients with advanced and severe cases of keratoconus still require corneal transplants [3].

Corneal cross-linking (CXL) has been used for more than 20 years, with the Dresden protocol being the most popular. The Dresden protocol involves removal of the corneal epithelium, administration of riboflavin eye drops, and administration of ultraviolet-A (UVA) light. Recently, various techniques have been developed as minimally invasive methods, including the epi-on method, supplemental oxygen administration, pulsed energy therapy, and iontophoresis. A recent review revealed that approximately 20 different CXL protocols have been developed [4].

One of these potential therapies for keratoconus that is still under investigation involves the use of violet light (VL)-emitting glasses (Tsubota Laboratory, Inc., Tokyo, Japan) [5]. KeraVio halted disease progression in patients with keratoconus, and its mechanism is similar to that of CXL. KeraVio treatment involves the use of eyeglasses with a 375 nm-wavelength VL source applied to the cornea, and patients wear eyeglasses daily without limitations. Corneal epithelial peeling in CXL has several potential risks, including ocular pain and corneal infection after surgery. We are still developing KeraVio treatment and have confirmed that low-intensity VL irradiation enhances the corneal elastic modulus by focusing on endogenous riboflavin in the human corneal stroma without the need to administer riboflavin eye drops [6]. Our hypothesis is that it would be possible to confirm the effects of KeraVio treatment without administering drugs. Physiological riboflavin in the human corneal stroma itself may affect corneal stiffness via VL irradiation.

This study aimed to report the clinical outcomes of KeraVio with VL irradiation without the administration of riboflavin drops in patients with progressive keratoconus.

## 2. Methods

We performed a prospective, four-center, randomized controlled study to assess the efficacy outcomes of KeraVio treatment without the administration of riboflavin drops. Institutional review board approval was obtained. The study adhered to the tenets of the Declaration of Helsinki. The study participants provided written informed consent. This study was approved by the Review Board at Shinanosaka Clinic and registered in the Japan Registry of Clinical Trials (jRCT): jRCTs032190267.

### 2.1. Inclusion and Exclusion Criteria

The inclusion criteria were as follows: male or female sex; any race or ethnicity; an age of 7 years or older; and a diagnosis of keratoconus as documented by topography or tomography. The subjects were also required to have experienced progression within 6 months before baseline to receive KeraVio treatment, as defined by one or more of the following: (1) an increase of ≥0.50 diopters (D) in the maximum keratometry value (Kmax); (2) an increase of ≥0.50 D in cylinder power on subjective manifest refraction; (3) an increase of ≥0.50 D in myopia on subjective manifest refraction; and (4) a decrease of ≥5 μm in the thinnest corneal thickness. Contact lenses were removed for a period before each visit to avoid changes in corneal shape. The period was 3 weeks for rigid gas-permeable lenses and 1 week for soft contact lenses. The combination of contact lenses and KeraVio treatment was limited to VL-transmitting lenses [7].

The exclusion criterion included a history of corneal surgery, including intracorneal ring segment surgery. Subjects who may have become candidates for corneal transplantation during the observation period were excluded.

### 2.2. KeraVio Treatment

The subjects were randomly assigned to the KeraVio with VL irradiation group or the control group, with a 2:1 ratio of trial to control patients. In the KeraVio with VL irradiation group, the subjects wore VL-emitting glasses [5], and their corneas were aligned and exposed to VL (375 nm) for 4.5 h daily for 6 months. Before each treatment, the desired irradiance of 0.31 mW/cm^2^ was verified with a UVA meter (LaserMate-Q; LASER 2000, Wessling, Germany) at a distance of 1.2 cm from the cornea and, if necessary, regulated with a potentiometer. The irradiance of the VL used in KeraVio was close to the physiological irradiance to which humans are exposed from sunlight. The concept of KeraVio is natural CXL using sunlight. The annual average VL irradiance in Tokyo is 0.31 mW/cm^2^, on the basis of our measurement data and the climate statistics available in the “Statistical Observations of Prefectures” by the Statistical Bureau of Japan [8]. To avoid the cytotoxic threshold of the corneal endothelium, which is 0.36 mW/cm^2^, we applied a VL intensity of 0.31 mW/cm^2^. With respect to the control group, the participants could have readily noticed if the eyeglasses emitted no light. Therefore, although the frames were identical in both groups, we made pseudoplacebo glasses for the control group with a minimal amount of VL irradiance (<0.01 mW/cm^2^) to maintain the double-blind nature of the study. The participants were masked by removing all eyeglass labels before they received the glasses [8]. The total energy doses of VL using eyeglasses for 6 months in the KeraVio with VL irradiation group and the control group were 904.0 and 29.2 J/cm^2^, respectively.

If both eyes per subject met the inclusion criteria, only the more severely affected eye was selected for treatment, after which a randomization procedure was performed for the two groups. The eye that was not treated was not exposed to VL by shielding the LED light source in the glasses.

### 2.3. Outcome Measures

**Tomography.** Tomographic data were obtained via anterior segment optical coherence tomography (AS-OCT) (CASIA^TM^, Tomey Corporation, Nagoya, Japan) at baseline and at 1, 3, and 6 months after KeraVio treatment. For quantification of keratometric parameters, the minimum corneal thickness and stromal demarcation line (DL) identified by the AS-OCT system were analyzed. Kmax was chosen as the primary efficacy outcome because it measures a salient feature of corneal ectasia, that is, the steepness of ectatic tomographic distortion. The identification of the DL via the AS-OCT scan was evaluated by two independent observers 1 month after treatment, as analytically determined in our previous studies [9,10].

**Visual acuity and refraction.** Uncorrected distance visual acuity (UDVA), corrected distance visual acuity (CDVA), and manifest refraction spherical equivalent (MRSE) were measured at baseline and at 1, 3, and 6 months after KeraVio treatment. Visual acuity measurements were obtained as the logarithm of the minimal angle of resolution (logMAR) units via a Landolt C chart.

In terms of the safety indices of VL in eyes, these indices were verified in our previous clinical trial, and we did not include these items from the present study.

### 2.4. Statistical Analysis

All the statistical analyses were performed via SPSS (SPSS Inc., Chicago, IL, USA) and MedCalc (MedCalc Software Ltd., Ostend, Belgium). Sample size calculation was performed via PASS 2008 software (NCSS, Kaysville, UT, USA). The outcome measures are reported as the mean ± standard deviation. The normality of all the data samples was first checked via the Kolmogorov-Smirnov test. The Mann-Whitney U test was used to compare the data between the two groups. A *p*-value of <0.05 was considered statistically significant. The sample size (*n* = 18:8, in each group) in this study offered 92% statistical power at the 5% level to detect a 0.6-D difference in Kmax between the two groups when the standard deviation of the mean difference was 0.4 D.

## 3. Results

### 3.1. Subject Demographics

Eighteen eyes from 18 patients were treated with KeraVio without the administration of riboflavin drops. Additionally, eight eyes of 8 patients were included in this study as a control group. The participant demographics are presented in Table 1. At baseline, the Kmax values in the KeraVio with VL irradiation group did not differ from those in the control group (*p* = 0.816), but age significantly differed (*p* = 0.030). All patients remained in the study through the 6-month follow-up.

### 3.2. Corneal Parameters

Table 2 shows the changes in Kmax and the thinnest corneal thickness values from baseline to the 6-month observation period after treatment. No significant differences were detected in the Kmax or thinnest corneal thickness between the two groups during the 6-month observation period. The mean changes in Kmax from baseline to 6 months in the KeraVio treatment with VL irradiation and control groups were 0.94 ± 2.65 D and 1.76 ± 2.75 D, respectively (*p* = 0.705). Similarly, the mean changes in the thinnest corneal thicknesses were −6.44 ± 19.16 μm and −0.50 ± 2.95 μm, respectively (*p* = 0.029).

The corneal stromal DL was identified in only one eye (5.6%) by both examiners in the KeraVio with VL irradiation group at 1 month. No DL was found in the control group.

### 3.3. Visual Acuity and Refraction

Table 3 shows the changes in CDVA, UDVA, and MESE from baseline to the 6-month observation period after treatment. No significant differences in these parameters were detected between the two groups during the 6-month observation period. The mean changes in CDVA from baseline to 6 months in the KeraVio with VL irradiation and control groups were 0.03 ± 0.13 logMAR and 0.04 ± 0.12 logMAR, respectively (*p* = 0.616). Similarly, the mean changes in UDVA were −0.09 ± 0.42 logMAR and 0.02 ± 0.30 logMAR, respectively (*p* = 0.404).

The mean changes in MRSE from baseline to 6 months in the KeraVio with VL irradiation and control groups were 0.91 ± 2.40 D and 0.03 ± 0.53 D, respectively (*p* = 0.304).

## 4. Discussion

In the present study, no significant differences in Kmax, vision, or refraction were observed between the group that received KeraVio treatment with VL irradiation without the administration of riboflavin drops and the control group during the 6-month observation period. Therefore, no differences were found between patients who did and did not receive VL irradiation in terms of the clinical outcomes of keratoconus. VL irradiation alone likely does not halt keratoconic progression, and the administration of riboflavin might be necessary to achieve significant efficacy in KeraVio treatment. The change in the thinnest corneal thickness was greater in the VL group than in the control group, but the interpretation of this result is difficult. This may be within the margin of error, or the reliability of its measurement may not be valid. We focused on the results of Kmax, vision, and refraction. On the other hand, in ex vivo porcine corneas, the KeraVio without riboflavin (VL irradiation only) and standard CXL groups presented significantly greater elastic moduli than the normal control group did, whereas no significant difference between the VL-only group and the CXL group was found [11]. These findings suggest that treatment involving only VL irradiation to the cornea is insufficient to inhibit the progression of keratoconus and that the administration of riboflavin eye drops might be needed to ensure the efficacy of KeraVio.

To confirm the effectiveness of KeraVio and CXL for keratoconus, it is important to confirm the presence or absence of the DL. In the present study of KeraVio treatment with VL irradiation alone, the DL was confirmed in only one eye of 18 patients (5.6%). On the other hand, in our previous clinical trial in which 0.05% flavin adenine dinucleotide (FAD) (Santen Pharmaceutical Co., Ltd., Osaka, Japan) eye drops were used in combination with KeraVio treatment, DL was observed in 16 eyes of 20 patients (80%) [5]. FAD functions as a coenzyme of riboflavin [12,13]. The mean Kmax value of the KeraVio-treated eyes decreased by 0.81 D at 6 months, suggesting that FAD or riboflavin eye drops are essential for the cessation of keratoconus progression [5]. In Japan, FAD eye drops have long been used to treat keratitis, but owing to the influence of pharmaceutical regulations, the distribution of the active pharmaceutical ingredient was suspended in 2022. We are still looking for ways to provide KeraVio treatment without the use of FAD eye drops, but the combination of VL irradiation and FAD eye drops was confirmed to be a prerequisite for efficacy in this clinical trial. We plan to create a new system to supply FAD eye drops or develop a protocol for oral riboflavin supplementation. Photocrosslinking via riboflavin and VL has been used to modify the collagenous matrix for ophthalmic applications [14].

Owing to technical limitations, it is difficult to quantify endogenous riboflavin in the corneal stroma of patients. We started this study in 2020 with the premise that endogenous riboflavin in the corneal stroma exists simultaneously in normal eyes in patients with keratoconus. However, a recent study by Sozer et al. [15]. reported that blood riboflavin levels were significantly lower in keratoconic eyes than in normal eyes. This may suggest that KeraVio treatment with VL alone was not effective in preventing the progression of the disease due to the low concentration of endogenous riboflavin in the corneal stroma in patients with keratoconus. Endogenous riboflavin may be present at low concentrations in the corneal stroma of keratoconus patients, so we believe that riboflavin administration is a prerequisite for successful treatment. We are currently investigating whether the preferred route of riboflavin administration should include the use of FAD (riboflavin) eye drops or oral riboflavin.

As a limitation of the present study, the sample size was not large; this was a pilot study conducted to evaluate the efficacy of KeraVio treatment with VL irradiation and without the administration of FAD drops. Considering the minimization of risk and the sufficient sample size, the sample size of this study was set at 26 patients in total. Another limitation was the short observation period. Although long-term follow-up was appropriate for determining the therapeutic effect of KeraVio, the Review Board Committee conducted a six-month observation period to minimize the progression of the disease in patients. The design of this study was a randomized controlled trial, and confounding factors such as age and sex were not considered when allocating subjects to the two groups. In addition, the subjects and researchers were double-blinded, so after this study was completed, it was discovered that there was an unbalanced allocation of age and sex.

The current study was multicenter, and the research budget was limited; therefore, this study did not evaluate keratocyte phenotypes or potential inflammation. We are conducting an evaluation of these parameters in terms of corneal morphology in future clinical studies of KeraVio treatment.

In conclusion, based on our 6-month follow-up results, no differences were found between patients who did and did not receive VL irradiation in terms of the clinical outcomes of keratoconus patients. VL irradiation alone likely did not halt keratoconic progression, and the administration of riboflavin was necessary.

## Figures and Tables

**Table 1:** Demographics of patients in the KeraVio with violet light irradiation group.

	KeraVio with VL Irradiation	Control	* *p*-Value
Eyes/patients (n)	18/18	8/8	n/a
Age (years)	28.56 ± 11.96	40.25 ± 11.72	0.030
Sex (female/male) (n)	14/4	6/2	n/a
Kmax (diopters)	56.17 ± 9.18	56.18 ± 8.35	0.816

* Compared with the control group. n/a = not applicable.

**Table 2:** Changes in corneal parameters over time.

	Baseline	1 Month	3 Months	6 Months	Change from Baseline to 6 Months
**Kmax (D)**					
KeraVio with VL irradiation	56.17 ± 9.18	55.75 ± 8.93	55.88 ± 9.43	57.11 ± 10.17	0.94 ± 2.65
Control	56.18 ± 8.35	56.43 ± 10.04	57.29 ± 10.18	57.23 ± 7.85	1.76 ± 2.75
* *p*-value	0.816	0.816	0.600	0.624	0.705
**Thinnest corneal thickness (** **μm)**					
KeraVio with VL irradiation	429.47 ± 63.54	428.88 ± 64.45	424.24 ± 64.84	422.24 ± 67.85	−6.44 ± 19.16
Control	415.63 ± 77.14	408.63 ± 80.66	417.63 ± 80.57	417.17 ± 79.10	−0.50 ± 2.95
* *p*-value	0.600	0.462	0.641	0.753	0.029

* Compared with the control group. D = diopters.

**Table 3:** Changes in visual acuity and refraction over time.

	Baseline	1 Month	3 Months	6 Months	Change from Baseline to 6 Months
**Corrected distance visual acuity (logMAR)**					
KeraVio with VL irradiation	0.20 ± 0.42	0.25 ± 0.48	0.27 ± 0.50	0.24 ± 0.46	0.03 ± 0.13
Control	0.33 ± 0.41	0.31 ± 0.39	0.39 ± 0.46	0.37 ± 0.49	0.04 ± 0.12
* *p*-value	0.534	0.728	0.388	0.604	0.616
**Uncorrected distance visual acuity (logMAR)**					
KeraVio with VL irradiation	0.84 ± 0.59	0.82 ± 0.63	0.80 ± 0.69	0.74 ± 0.61	−0.09 ± 0.42
Control	0.98 ± 0.47	0.87 ± 0.73	0.90 ± 0.72	1.07 ± 0.80	0.02 ± 0.30
* *p*-value	0.600	0.863	0.918	0.352	0.404
**Manifest refraction spherical** **equivalent** **(D)**					
KeraVio with VL irradiation	−6.57 ± 7.29	−6.54 ± 7.21	−6.15 ± 6.89	−5.67 ± 7.50	0.91 ± 2.40
Control	−7.73 ± 7.42	−7.67 ± 7.30	−7.97 ± 6.83	−7.04 ± 7.70	0.03 ± 0.53
* *p*-value	0.999	0.864	0.682	0.680	0.304

* Compared with the control group. logMAR = logarithm of the minimal angle of resolution. D = diopters.

## Data Availability

Data are available upon reasonable request.

## References

[1] Rabinowitz Y.S. (1998). Keratoconus. Surv. Ophthalmol..

[2] Beltaief O., Farah H., Kamoun R., Ben Said A., Ouertani A. (2003). La greffe de cornéee chez l’enfant [Penetrating keratoplasty in children]. Tunis Med..

[3] Reeves S.W., Stinnett S., Adelman R.A., Afshari N.A. (2005). Risk factors for progression to penetrating keratoplasty in patients with keratoconus. Am. J. Ophthalmol..

[4] Wu D., Lim D.K., Lim B.X.H., Wong N., Hafezi F., Manotosh R., Lim C.H.L. (2021). Corneal Cross-Linking: The Evolution of Treatment for Corneal Diseases. Front. Pharmacol..

[5] Kobashi H., Torii H., Toda I., Kondo S., Itoi M., Tsubota K. (2021). Clinical outcomes of KeraVio using violet light: Emitting glasses and riboflavin drops for corneal ectasia: A pilot study. Br. J. Ophthalmol..

[6] Kobashi H., Yunoki S., Kato N., Shimazaki J., Ide T., Tsubota K. (2021). Evaluation of the Physiological Corneal Intrastromal Riboflavin Concentration and the Corneal Elastic Modulus After Violet Light Irradiation. Transl. Vis. Sci. Technol..

[7] Torii H., Kurihara T., Seko Y., Negishi K., Ohnuma K., Inaba T., Kawashima M., Jiang X., Kondo S., Miyauchi M. (2017). Violet Light Exposure Can Be a Preventive Strategy Against Myopia Progression. eBioMedicine.

[8] Torii H., Mori K., Okano T., Kondo S., Yang H.Y., Yotsukura E., Hanyuda A., Ogawa M., Negishi K., Kurihara T. (2022). Short-Term Exposure to Violet Light Emitted from Eyeglass Frames in Myopic Children: A Randomized Pilot Clinical Trial. J. Clin. Med..

[9] Kymionis G.D., Grentzelos M.A., Plaka A.D., Tsoulnaras K.I., Diakonis V.F., Liakopoulos D.A., Kankariya V.P., Pallikaris A.I. (2014). Correlation of the corneal collagen cross-linking demarcation line using confocal microscopy and anterior segment optical coherence tomography in keratoconic patients. Am. J. Ophthalmol..

[10] Kymionis G.D., Tsoulnaras K.I., Grentzelos M.A., Plaka A.D., Mikropoulos D.G., Liakopoulos D.A., Tsakalis N.G., Pallikaris I.G. (2014). Corneal stroma demarcation line after standard and high-intensity collagen crosslinking determined with anterior segment optical coherence tomography. J. Cataract. Refract. Surg..

[11] Kobashi H., Yano T., Tsubota K. (2023). Combination of violet light irradiation and collagenase treatments in a rabbit model of keratoconus. Front. Med..

[12] Sakata R., Sakisaka T., Matsuo H., Miyata K., Aihara M. (2016). Effect of Travoprost and Nonsteroidal Anti-Inflammatory Drug on Diurnal Intraocular Pressure in Normal Subjects with Low-Teen Baseline Intraocular Pressure. J. Ocul. Pharmacol. Ther..

[13] Sakata R., Sakisaka T., Matsuo H., Miyata K., Aihara M. (2016). Time Course of Prostaglandin Analog-related Conjunctival Hyperemia and the Effect of a Nonsteroidal Anti-inflammatory Ophthalmic Solution. J. Glaucoma.

[14] Lai J.Y. (2014). Photo-cross-linking of amniotic membranes for limbal epithelial cell cultivation. Mater. Sci. Eng. C Mater. Biol. Appl..

[15] Sozer O., Ozalp O., Atalay E., Demir S.S., Alatas İ.O., Yildirim N. (2023). Comparison of blood levels of vitamin B12, folic acid, riboflavin, and homocysteine in keratoconus and healthy subjects. J. Cataract. Refract. Surg..

